# What can Adorno’s understanding of aesthetic experience offer for the health and medical humanities?

**DOI:** 10.1007/s11019-025-10316-0

**Published:** 2025-12-23

**Authors:** Anna Ilona Rajala

**Affiliations:** 1 University of the Arts Helsinki, Theatre Academy, Helsinki, Finland; 2 Tampere University, Faculty of Management and Business, Politics Unit, Tampere, Finland

**Keywords:** Adorno, Aesthetic experience, Art, Critical theory, Health humanities, Medical humanities

## Abstract

The concept of aesthetic experience is essential to understand the ways people interact with, immerse in, and interpret aesthetic objects, such as artworks, literature, or natural beauty. Aesthetic experience is also important within the health and medical humanities in explaining the effects and benefits of engaging with art, but philosophical perspectives on the concept within the field are surprisingly scarce. This article addresses this research gap by delving into one such perspective in the Western philosophical tradition that draws on the aesthetic theory of Theodor W. Adorno. I argue that Adorno’s understanding of aesthetic experience offers valuable support for the critical turn in the health and medical humanities, which seeks to move beyond the clinical encounter toward the broader societal context of health and illness. Adorno’s relevance lies in his vehement argumentation against an instrumentalising understanding of aesthetic experience. According to Adorno, the experiencing subject ought not to be understood simply as someone who judges between good and bad art, beautiful and ugly, or aesthetic and nonaesthetic, because such judgements can ultimately be reduced to arguments about art as having or not having exchange value. Instead, aesthetic experience is something more profound: a mode of knowledge rarely accessible through other means, in which the experiencing subject enters into the artwork and activates its own subjectivity. This argument offers the health and medical humanities insight into how art might be approached in relation to its potentially transformative role in society.

## Introduction

The philosophical study of art and beauty is at least as old as philosophy itself, and aesthetic experience is a central concept to understand the ways people interact with, immerse in, and interpret aesthetic objects, such as artworks, music, literature, natural beauty, and even mathematical theorems. In Western philosophy from the 18th century onwards, the concept of aesthetic experience has been variously described as the intentional and engaged experience of the beauty—or sometimes the sublimity—of an object, which can be directly perceptual (e.g., seen, heard), sensorily imagined (e.g., imagined sensory experiences when reading poetry), or more intellectual and abstract (e.g., in the case of mathematical theorems) (Peacocke 2023). In contemporary research, aesthetic experience forms a multidisciplinary epistemological boundary object: it is one object of study shared by several disciplines and social contexts that adhere to differing views of knowledge (Liljefors 2020, pp. 206–7). Therefore, there is no consensus on how exactly aesthetic experience ought to be defined. It is widely recognised, however, that aesthetic experience is qualitatively different from everyday experience and has remained central to humanity throughout history, even as its forms and meanings have been historically contingent and shaped by cultural and social contexts (Liljefors 2020, p. 210; Marković 2012, p. 1; Pearce et al. 2016; Tomlin 2007).

Aesthetic experience is also an epistemological boundary object within the health and medical humanities, and it has been explored in different contexts (see e.g., Ahlzén 2007; Liatsos 2019; Spiegel and Spencer 2016). Both encountering patients and experiencing illness are fundamentally aesthetic, at least in the wider sense of the Greek *aisthēsis*, as etymologically and conceptually related to senses and perception (Vichnar and Armand 2017). On the one hand, from a clinical perspective, engaging with and experiencing aesthetic objects has been argued and observed to enhance clinicians’ narrative competences, such as empathy and patient-centred care (Arntfield et al. 2013; Charise 2020; Chu, Wen, and Lin 2020; Charon 2008). There is also a likeness between aesthetic experience in encountering artworks and in encountering people at the clinic, and aesthetic interpretation of art can thus evoke new perspectives and experiential affordances, creating new worlds of meaning (Kelly 2024; see also Valtonen and Renko 2025). Moreover, as Thompson (2023, p. 1) argues, caring—whether formal care provided by healthcare practitioners or informal care practiced in everyday life—can be considered an aesthetic practice, because “it has a certain craft and involves the creation of sensory, embodied experiences”. From the perspective of experiencing illness, on the other hand, engaging therapeutically in experiencing art has been shown to have effects for physical, psychological, and existential wellbeing (Mastandrea, Fagioli, and Biasi 2019; Malchiodi 2012; Sajnani and Fietje 2023). Numerous biographical, autobiographical, and fictional works on the experience of illness also demonstrate the power of aesthetic expression and aesthetic experience within these narratives (see e.g., Clark 2020; Gilman 2004; Rieff 2008).

Aesthetic experience is often thought to be fundamentally subjective (Liljefors 2020, p. 209). The experiencing subject as the ultimate locus of aesthetic experience has also been challenged in philosophy, for example, by German Romantics such as Johan Gottfried Herder in the 19th century (see Ross 2017), and later in the works of philosophers such as John Dewey, Hans-Georg Gadamer, and Richard Shusterman (see e.g., Dewey 2005; Gadamer 1986; Shusterman 2008). Each of these philosophers, as well as many other thinkers, can offer the health and medical humanities helpful perspectives into aesthetic experience. For example, for Gadamer, aesthetic experience offers a mode of understanding that reveals truths inaccessible through scientific methods, while Dewey argues that art is not just an object but a lived experience, a process. Drawing on Dewey’s work, Shusterman emphasises the importance of the body in aesthetic experience as actively engaged rather than a passive vessel. The purpose of this article, however, is to delve deeper into one philosophical perspective among many fruitful options: the aesthetic theory of the German philosopher, sociologist, and musicologist Theodor W. Adorno.

Adorno’s aesthetic theory, which had been gestating since before the Second World War and finally culminated in the posthumously published *Aesthetic Theory* in 1970, offers valuable insight for the analysis of aesthetic experience within the health and medical humanities. It has a lot to offer, as I aim to show, particularly in terms of understanding the role of art (understood here as encompassing the full spectrum of artistic practices) beyond its instrumental use and emphasising its transformative potential in society. For Adorno, aesthetic experience is a dialectical relationship between subject and object, but more importantly, it is also a dialectical relationship between art and social reality. Adorno argued vehemently against an instrumentalising understanding of the experiencing subject as someone judging between good and bad art, beautiful and ugly, or aesthetic and nonaesthetic. Such judgements can ultimately be reduced to arguments about whether art has exchange value and, consequently, whether artworks serve merely as commodities within society. Instead, aesthetic experience is something more profound: it is a mode of knowledge inaccessible by any other means, in which the subject enters the work of art and activates art’s own subjectivity. Aesthetic experience, then, is not simply about making an aesthetic judgement. Rather, the aesthetic object forces the experiencer into a reflection that potentially transforms their very understanding of themselves and offers a site for societal critique. To put it differently, we do not simply judge art as art-consuming individuals; it is art that judges us and the society in which we live.

Despite being an important concept within the health and medical humanities to describe what happens between the experiencing subject and the experienced object, philosophical research on aesthetic experience remains surprisingly scarce in the field (see e.g., Adamson 2017; Cooper 2024; Liljefors 2020). To address this gap, I begin by introducing Adorno’s understanding of aesthetic experience, nonidentity, and the subject–object dialectic. These help to explain why Adorno argued against aesthetic experience being merely instrumental. I then explore Adorno’s notion of aesthetic experience in greater depth by clarifying what he means by art possessing subjectivity, which inevitably brings Adorno’s focus to society. Finally, to conclude, I consider the implications of engaging with the political for the health and medical humanities. My discussion contributes to the critical turn within the health and medical humanities, which seeks to widen the focus and scale of ‘the medical’ beyond the clinical encounter toward understanding how health and illness are constituted within their complex societal context (Viney et al. 2015; Whitehead et al. 2016). Adorno’s understanding of aesthetic experience offers valuable insight for the health and medical humanities into how art might be approached in relation to its potentially transformative role in society.

## Against mere instrumentality of aesthetic experience

The concept of aesthetic experience tends to evoke an image of a subject that is the final source of aesthetic judgement and the locus of aesthetic experience: when I visit art galleries, read books, or listen to music, I perceive artistic objects through my senses, and make aesthetic judgements about liking or disliking them. Thus, the experience seems to occur in me. Western philosophical aesthetics has a long history of such thinking. As Jay (2005, p. 158) explains, “in virtually all of its manifestations [in the history of philosophy], aesthetic experience meant a privileging of the subject, whether contemplative, productive, or self-fashioning, over the art object”. In philosophical aesthetics, aesthetic experience can be understood as subjective in the sense of being experienced by a subject, but it is also widely recognised to be grounded in the objective qualities of that which is experienced aesthetically (Hagman 2005, p. 15). In this sense, it is an embodied and affective reciprocal process between the experiencer and the experienced (Brinck 2018; Shusterman 2008; Stephens 2021). In other words, the experiencing subject and the experienced object depend on each other in the constitution of aesthetic experience. However, recognising the interdependence between subject and object does not yet reveal much about the power relations within this dynamic: what is the ‘use’ of aesthetic experience, and for whom—the subject, the object, or perhaps both?

Adorno is among those philosophers who challenge the understanding of aesthetic experience as ultimately reducible to the subject. Adorno is particularly critical towards Immanuel Kant’s influential aesthetics as the judgement of taste, the disinterested pleasure in the beautiful, which Kant discusses from the subjective perspective of receiving objects (e.g. Kant 2000, pp. 90–96; Adorno 2002, p. 163). Such subjective judgement, as Hohendahl (2010, p. 41) explains, “remains [in Adorno’s view] extrinsic and thereby fails to disclose the essence of the artwork”. Adorno departs from the Kantian view in that aesthetic experience requires real involvement in the artwork and remains, so to speak, inside the artwork rather than judging it from the outside or from above (Hohendahl 2010, p. 41). The key to understanding this lies in Adorno’s rather complicated subject–object dialectic, which prioritises the object of experience, because only by giving priority to the object—only by submitting to the object and its complexity instead of forcing it to submit to the subject’s interpretations—can the subject become truly and concretely involved in the artwork.

To better understand this dialectic, let us first pause for a moment at Adorno’s understanding of experience more generally. Adorno refers to a specifically dialectical notion of experience (*Erfahrung* in original German) that is indebted to the philosophies of both G.W.F. Hegel and Walter Benjamin. This dialectical notion, Jay (2005, p. 11) writes, “connotes a progressive, if not always smooth, movement over time, which is implied by the *Fahrt* (journey) embedded in *Erfahrung* and the linkage with the German word for danger (*Gefahr*)”. Since Jay’s genealogy of experience in Western thought is the most extensive exploration of the concept to date, let him explain further, in an emphatically Adornian vein:

However much we may construe experience as a personal possession—“no one can take my experiences away from me,” it is sometimes argued—it is inevitably acquired through an encounter with otherness, whether human or not. That is, an experience, however we define it, cannot simply duplicate the prior reality of the one who undergoes it, leaving him or her precisely as before; something must be altered, something new must happen, to make the term meaningful. (Jay 2005, p. 7)

While Adorno rarely used the word ‘otherness’ exactly, the key here to understanding experience as *Erfahrung* is the encounter with otherness—whether it is human, non-human, animate, or inanimate—that alters the experiencing subject. To put it differently, experience happens if and only if some change happens in the subject through encountering otherness—or, the object. If the encounter leaves the subject unaltered or indifferent, it was not *Erfahrung* that the subject went through, but something more superficial. This also applies to Adorno’s understanding of aesthetic experience: it was something other than aesthetic experience (in the sense of *Erfahrung*) if the subject is left the same as before.

Adorno departs from the Kantian subject-prioritising understanding of experience by reversing it. Kant maintained that the subject cannot experience the object as it is in itself. Rather, according to Kant (1998, 361–64), the object is grasped only as structured by the categories of mind—as constituted by the subject, or as Buck-Morss (1977, pp. 82–83) put it, “as something essentially identical to the subject”. The aim of Adorno’s thinking, which was remarkably consistent throughout his intellectual career spanning from the 1920s until his death in 1969, was to break such ‘identity thinking’ from within. So, instead of following identity thinking—meaning the need to positively identify, define, determine, categorise, and subjugate objects, and subsume their uniqueness and particularity under universal subjective concepts—Adorno’s *negative* dialectics seeks consciousness of the *nonidentical*. Adorno never defined the nonidentical, because definitions represent identity thinking par excellence, but he described it as, for example, the ineffable, the non-conceptual, the ungraspable, the concept’s other, the undissolvable, the unmastered, the concept’s untruth, the incomprehensible, the unknown, and that of which one cannot speak (see e.g., Adorno 1994, p. 108, 133–37, 147).

Consciousness of the nonidentical requires placing the experiential priority on the object, since prioritising the subject aligns with identity thinking, whereas prioritising the object enables immersion in the nonidentical. For Adorno, however, the priority of the object does not imply a one-way relationship between the subject and the object. Instead, they mutually mediate each other, but not as opposite poles in the movement of consciousness that constitutes experience. Rather, mediation means that it is impossible to discern any pure subjectivity or pure objectivity; both of them always contain traces of each other. The priority of the object, and here Adorno is worth quoting in length despite the complexity of his style of writing, also suggests that:

subject for its part is object in a qualitatively different, more radical sense than object, because object cannot be known except through consciousness, hence is also subject. What is known through consciousness must be something; mediation applies to something mediated. But subject, the epitome of mediation, is the ‘How’, and never, as contrasted to the object, the ‘What’ that is postulated by every conceivable idea for a concept of subject. Potentially, though not actually, objectivity can be conceived without a subject; but not likewise object without subjectivity. No matter how subject is defined, the existent being cannot be conjured away from it. If subject is not something, and “something” designates an irreducibly objective element, then it is nothing at all […] Object is also mediated; but, according to its own concept, it is not so thoroughly dependent upon subject as subject is dependent on objectivity. (Adorno 2005a, pp. 249–50)

Adorno argues here, in short, that even if the object should take precedence in experience, the relationship is not hierarchical: “there is nothing subjective that is not mediated, there is likewise nothing objective that is not mediated” (Adorno 2019, p. 124). For the subject, then, the priority of the object means that the subject is always also an object, or something extant, real, and material, rather than just the Kantian category-employing rational subject. The mediation of the object by the subject means that the object is determinate only through the subject; but, because the object depends on subjective reflection rather than being conceived in any pure objective form, “subjectivity becomes a moment that is held fast” (Adorno 2005a, p. 251). The subject, however, does not have absolute power over the object, but is rather “the agent, not the constituent, of object” (Adorno 2005a, p. 254). For the object, in turn, being mediated by the subject means that if the “subject has a core of object, then the subjective qualities in the object are all the more objective moment. For object becomes something at all only through being something determinate” (Adorno 2005a, p. 250).

In more practical terms, the priority of the object means casting a “long and uncoercive gaze upon the object” (Adorno 2005a, p. 130) which embodies for Adorno, as Richter (2019, p. 38) elaborates, a specific kind of comportment that thinks through the object, enabling the subject to snuggle up and tarry with the object, its singularities, its idiomaticities, and difference. Describing the priority of the object in these terms also explains Adorno’s understanding of aesthetic experience which, according to Adorno, cannot be grounded on subjectivity alone (Adorno 2002, p. 142), because any understanding of the subject’s aesthetic experience is deficient without the object—the experienced artwork—mediating it (Adorno 2002, p. 166).

So, where does aesthetic experience occur, and why is this question significant for the health and medical humanities? According to Adorno, aesthetic experience never arises solely within the subject or through the subject without an object; nor does the object become aesthetic (in the sense of *aisthēsis*) without an experiencing subject. However, for aesthetic experience to matter—to bring about a transformation in the subject in the sense of *Erfahrung*—art must not be judged externally or forced into the subject’s interpretative framework but rather approached from within: by giving priority to the object and allowing it to have its say, so to speak. Billington (2016, p. 44) echoes this in the health and medical humanities context: “literature can ‘think’ reality when ordinary human thought falls short; that a book can have thoughts humans *cannot* have”. The significance of this for the health and medical humanities lies in the shift in perspective from the interpreting and judging subject to the prioritisation of the object, which underscores the importance of aesthetic experience beyond its instrumental applications.

Aesthetic experience is sometimes—though not exclusively—framed within health, medical, and neuroscience contexts as a process of engaging with aesthetic objects or aesthetically pleasing works of art that evoke reflection, leading to humanising effects and predominantly positive observable outcomes (see e.g., Bittar, De Sousa, and Claramonte Gallian 2013; Kontson et al. 2015; Weigand, Moosmayer, and Jacobsen 2021). Such an approach tends to prioritise the subject: its pleasurable experience, its aesthetic judgement, and the impacts within it. In contrast, engaging with art by prioritising the object offers art a more profound and potentially transformative role—one that is not restricted to consuming the beautiful, pleasing, and immediately useful. As Liljefors (2020, p. 212) argues, while it is the *raison d’être* of the health and medical humanities to approach art in a more instrumental fashion, instrumentalisation is, at the same time, anathema to the study of art within the broader field of the humanities (see also Macnaughton 2011).

The debate over whether art should be appreciated for its own sake or assigned merely instrumental value has been ongoing for at least two centuries and it is not easily resolved (see Charise 2020; Liljefors 2020). However, this question is important to explore within the field of health and medical humanities, because reducing art to its social or economic utility undermines aesthetic experience. As Adorno argues, art provides powerful experiences precisely by standing in opposition to prevailing social realities. Liljefors (2020) echoes this in the context of health and medical humanities, suggesting that focusing on aesthetic experience itself already resists instrumentalisation, as it offers existential health rather than more specific health effects. If this is true, “then art activities in healthcare no longer appear a trivializing instrumentalization of art for external purposes, but rather a realization of the genuine essence of aesthetic experience” (Liljefors 2020, p. 216). It may also be argued, as Liatsos (2019) suggests, that aesthetic experience is already embedded in the practice of health and medical humanities. Or, to reframe this in line with Adorno’s aesthetic theory: if aesthetic experience as *Erfahrung* occurs, the effects of the aesthetic object acting upon the subject and their surroundings are far too profound to be reduced to their mere instrumental value.

## Art as a subject and an agent of societal critique

The claim that art plays a more profound and potentially transformative role beyond its instrumental use in society—and, by extension, in healthcare—warrants a closer examination of Adorno’s argument regarding art gaining subjectivity. Therefore, I now turn to the question: how is it possible for an artwork to acquire its own subjectivity as an object? Bearing in mind the subject–object dialectic and the priority of the object introduced above, aesthetic experience can be understood not as a passive response to art, but as an active experience of the particularity of objects—an encounter that alters the experiencing subject. For Adorno, aesthetic experience not only moves us away from the idea of absolute constitutive subjectivity, but it also forms an “unimpaired corrective of the reified consciousness that wants to claim totality” (Adorno 2002, p. 330; amended translation by Silberbusch 2018, p. 139). Here, the subjectivity of the artwork becomes visible as it challenges the absolute authority of the constitutive subject. Aesthetic experience can be thought of as a mode of knowledge “that allows us to enter in to works of art, to activate their own subjectivity rather than making them instruments of our own” (Silberbusch 2018, p. 139). This demands of the subject a stance that does not seek to dominate the object of its experience by projecting a meaning onto it but rather allows the object to speak for itself. Adorno (2017, pp. 25–26) explains:

I understand a work of art at the moment when […] I understand what it is itself saying as something it says to me, not as something I am projecting onto it, something that has come only from me. (Adorno 2017, pp. 25–26)

This is where Adorno’s argument becomes gripping: how can an inanimate object, such as a work of art, say something as itself and be that something it is saying to me? Adorno (2002, p. 193) argues that as we enter into and follow the artwork, the artwork absorbs us. It draws us inside itself, and so “forces us to accompany it on the paths it traces within itself” (Adorno 2017, p. 120). Or, as Silberbusch (2018, p. 139) explains, “[t]he artwork is not grasped by us, we are grasped by it”. This means that we do not simply judge art so much as art judges us by forcing us into a reflection that transforms our self-conception (Ross 2017, p. 138), which profoundly affects the ways in which we act in the world. As we nestle up to the artwork, cast a long and uncoercive gaze upon it, and follow its lead rather than immediately trying to rationalise it, we lose ourselves in it—and this affects us profoundly. This is why aesthetic experience is thought of as distinct from everyday experience: the artwork gains subjectivity because it interacts with us and affects us and also acts through us and our actions. However, for it to do so, we must give the priority to the object: to “let the ungraspable stand without subjugating it” (Silberbusch 2018, p. 139).

Adorno’s aesthetic theory is embedded in societal critique and philosophy. Art cannot be conceived as detached from society, and philosophy is the medium through which their relationship can be understood. For Adorno, both Marxist philosophy and leftist politics had failed to deliver on their promises. In his eleventh thesis on Feuerbach, Marx had claimed that “philosophers have hitherto only *interpreted* the world, when the point is to *change* it” (Marx 1977, p. 423). Adorno (2014, p. 58), however, turns Marx on his head, suggesting instead that the world probably has not changed because it has not been interpreted enough. In other words, if the point of philosophy is to change the world for the better, as Marx had claimed, then it must keep interpreting it to explain why societal change has not happened and is still not happening; and the point of philosophy is to create grounds for societal change through the critique of society. Indeed, for Adorno, the fact that the world has not changed is why philosophy is still urgently needed: “Philosophy, which once seemed obsolete, lives on because the moment to realize it was missed” (Adorno 1973, p. 3).

Adorno, however, does not rely solely on philosophical interpretation in his critique of society; he also emphasises the fundamental importance of art as a site of resistance to life under late capitalism—a life so distorted that a truly just existence can scarcely be imagined (Adorno 2005b, p. 39). Philosophy and art are mutually dependent: art needs philosophy to interpret it, to offer art a language, while “art is only able to say what it says by not saying it” (Adorno 2002, p. 72). Compared to philosophy, according to Adorno as Hohendahl (2010, p. 72) elaborates, art’s “lack of conceptual rigor […] comes across as an advantage since it can point to a truth content beyond the realm of rational discourse”. This does not imply that art must depict political themes or messages for it to be political. Rather, art is political precisely because it offers something qualitatively different from everyday experience and because it makes other kinds of modes of experience possible. As Ross (2017, p. 1) put it, art “challenges us to see the world with new eyes, to question conceptions of knowledge and truth, and to explore new possibilities of praxis”.

For Adorno, aesthetic experience is not merely the experience of artworks or other objects with aesthetic qualities; rather, it simultaneously describes the way in which an aesthetic object contains an experience of reality beyond what happens between the experiencing subject and the experienced object. In aesthetic experience, it is not only the subject that is altered, but the object also responds to that world in which the subject is embedded. In this sense, if art can gain something like subjectivity in dialectical aesthetic experience, then it is not only the subject that is affected but also, through the experientially altered subject, the subject’s social surroundings. Aesthetic experience, as Ross (2017, p. 1) notes, is “an indispensable way to gain critical insight into the social and political context of our lives”. This notion is important for Adorno because it asserts that artworks can have actual social and societal significance rather than being merely objects to be consumed for aesthetic pleasure.

However, aesthetic experience—or philosophical critique for that matter—cannot guarantee a transformed society. The only thing that Adorno believes can be guaranteed is hope: art offers glimpses of hope and redemptive modes of knowledge of the self and society that transcend both the current social reality and its aesthetic representation (Adorno 1973, 2002; Hohendahl 1995, pp. 71–72). Art is not social because it simply represents reality. Rather, it becomes social by standing in opposition to society—as something wholly other and autonomous, qualitatively distinct from social reality, yet still socially conditioned, created, and experienced by socially conditioned subjects (Adorno 2002, p. 225; Hohendahl 2010; Zuidervaart 1991). Because of this “tension-ridden double trajectory” (Hohendahl 2010, p. 221), it is not the artwork as a solipsistic object of enjoyment that offers hope for redemption of society; rather, redemption can only be hoped for through an intense aesthetic experience. So, in the case of art, redemption means turning artistic objects from objects of our enjoyment into media of reflection—and this, fundamentally, is what the health and medical humanities aim to achieve.

## Conclusion: toward critical health and medical humanities

How does Adorno’s aesthetic experience matter for the health and medical humanities? The argument that art is an acting subject in societal critique is relevant to the field, particularly in supporting its critical turn that widens the scope beyond the “primal scene” of the clinic to the complex context of society (see e.g., Viney et al. 2015; Whitehead and Woods 2016; see also Charon et al. 2021). As various other fields within the humanities—such as narrative theory—have also shifted toward politically, socially, and environmentally conscious directions aimed at promoting social justice (see Mäkelä & Meretoja 2022; Ovaska 2024), what has become to matter is not only the aesthetic objects themselves but also how we engage with them. More than two decades ago, Charon (2001), the founder of narrative medicine, noted that:

Despite—or, more radically, because of—economic forces that shrink the time available for conversation and that limit the continuity of clinical relationships, medicine has begun to affirm the importance of telling and listening to the stories of illness. As practice speeds up, physicians need all the more powerful methods for achieving empathic and effective therapeutic relationships. Narrative skills can provide such methods to help physicians join with their patients, honoring all they tell them. (Charon 2001)

Here, the very rationale for developing the narrative medicine approach is political: time and resource constraints, and their dehumanising burden on the healthcare workforce, serve as reasons to bring art into healthcare, where it can function as an antidote for cynicism.

However, the focus of the health and medical humanities should not rest solely on the individual practitioner who becomes more empathetic and less cynical through engagement with art. While this is undoubtedly important, it is insufficient when the broader context in which they practice remains unfavourable. Globally, neoliberal policies, austerity politics, shifting economic and political climates, global health crises, insecurity and war, and forced displacement have made healthcare environments increasingly inhospitable—not only for those in need of care but also for the professionals providing it (see e.g., Ameso and Prince 2022; Inhorn and Volk 2021; Mohiuddin 2023; Tarnas et al. 2024; Whiley and Grandy 2022; World Health Organization 2022). Within such political contexts, beyond its effects at the level of individual practitioners, art can also function as a medium for reflecting the societal conditions that give rise to practitioner cynicism in the first place.

But is this not just another means-to-an-end logic that risks instrumentalising art? And if so, is it possible to transcend it? Aesthetic experience, in Adorno’s terms, seeks to resist the reduction of art to utility by inviting reflection, embracing ambiguity and nonidentity, and allowing for immersion in the artwork—through the long and uncoercive gaze—with sufficient time for contemplation. Art’s subjectivity strongly challenges the notion that the health and medical humanities can proceed on purely instrumental grounds (Liljefors 2020). Aesthetic experience is simply too complex to be captured by instrumental reason. In this sense, art—alongside other qualitative approaches—can rightfully claim its place as a legitimate means through which to study health and medicine, rather than merely as a tool appended to the ‘hard’ clinical sciences to humanise them. While art is employed in healthcare contexts for various beneficial and humanising purposes—sometimes predetermined, sometimes unforeseen—Adorno’s thinking points to an additional, and perhaps far more significant, role: engaging with art allows for building knowledge of the self and society that can scarcely be cultivated anywhere else, often offering only gestating glimpses of hope that prevailing injustices may be overcome. As Adorno (2002, p. 136) famously writes: “Art is the ever broken promise of happiness”. However, strategic arguments that emphasise the instrumental value of art can also be useful, even necessary—particularly in contexts saturated with policies that threaten anything and everything not demonstrably linked to economic or utilitarian outcomes.

Understanding aesthetic experience as dialectical is not a panacea. What it can offer is something qualitatively different from the political, economic, and ideological conditions in which most healthcare systems operate today—a site of refuge, hope, and redemption, as Adorno might say, from which it becomes possible to approach the health and medical humanities critically. Precisely because art is not commensurate with the social context that seeks to render it obsolete on economic and political grounds, it holds radical, even utopian potential within the health and medical humanities for imagining an otherwise world.

## References

[CR1] Adamson, Veronica M. F. 2017. *The aesthetic experience of dying: The dance to death*. Abingdon: Routledge.

[CR2] Adorno, Theodor W. 2002. *Aesthetic theory*. London: Continuum.

[CR3] Adorno, Theodor W. 1973. *Negative Dialectics*. London: Routledge.

[CR4] Adorno, Theodor W. 1994. *Hegel: Three Studies*. Cambridge, MA: MIT Press.

[CR5] Adorno, Theodor W. 2005a. *Critical Models: Interventions and Catchwords*. New York: Columbia University Press.

[CR6] Adorno, Theodor W. 2005b. *Minima Moralia: Reflections from Damaged Life*. London: Verso.

[CR7] Adorno, Theodor W. 2014. *Lectures on Negative Dialectics: Fragments of a Lecture Course 1965/1966*. Cambridge: Polity.

[CR8] Adorno, Theodor W. 2017. *Aesthetics*. Cambridge: Polity.

[CR9] Ahlzén, Rolf. 2007. Medical humanities – Arts and humanistic science. *Medicine, Health Care and Philosophy* 10 (4): 385–393. 10.1007/s11019-007-9081-3. 17624812 10.1007/s11019-007-9081-3

[CR10] Ameso, Edwin Ambani, and Prince Ruth Jane. 2022. Striking health workers: Precarity and healthcare in neoliberal Kenya. *Anthropology Today* 38(4):11–14. 10.1111/1467-8322.12742

[CR11] Arntfield, Shannon L., Kristen Slesar, Jennifer Dickson, and Rita Charon. 2013. Narrative medicine as a means of training medical students toward residency competencies. *Patient Education and Counseling* 91(3):280–286. 10.1016/j.pec.2013.01.014 23462070 10.1016/j.pec.2013.01.014 PMC3992707

[CR12] Billington, Josie. 2016. *Is Literature Healthy? The Literary Agenda*. Oxford: Oxford University Press.

[CR13] Bittar, Yuri, Maria Sharmila Alina. De Sousa, and Dante Marcello Claramonte. Gallian. 2013. The aesthetic experience of literature as means for health humanisation in Brazil: The Laboratory of Humanities from EPM (UNIFESP). *The International Journal of Health, Wellness, and Society* 3 (1): 25–42. 10.18848/2156-8960/CGP/v03i01/41039.

[CR14] Brinck, Ingar. 2018. Empathy, engagement, entrainment: The interaction dynamics of aesthetic experience. *Cognitive Processing* 19 (2): 201–213. 10.1007/s10339-017-0805-x. 28391411 10.1007/s10339-017-0805-x PMC5976699

[CR15] Buck-Morss, Susan. 1977. *Origin of Negative Dialectics*. Hassocks: The Harvester Press.

[CR16] Adorno, Theodor W. 2019. *Philosophical Elements of a Theory of Society*. Cambridge: Polity.

[CR17] Charise, Andrea. 2020. On applying Art and humanities in austere times. In *The Routledge companion to health humanities*, ed. Paul Crawford, Brian Brown, and Andrea Charise. 18–26. London: Routledge.

[CR18] Charon, Rita, Craig Irvine, Aaron Ngozi Oforlea, Edgar Rivera Colón, and Cindy Smalletz, Maura Spiegel. 2021. Racial justice in medicine: Narrative practices toward equity. *Narrative* 29(2):160–177. 10.1353/nar.2021.0008

[CR19] Charon, Rita. 2001. Narrative medicine: A model for empathy, reflection, profession, and trust. *JAMA* 286 (15): 1897. 10.1001/jama.286.15.1897. 11597295 10.1001/jama.286.15.1897

[CR20] Charon, Rita. 2008. *Narrative medicine: Honoring the stories of illness*. Oxford: Oxford University Press.

[CR21] Chu, Shao-Yin, Chin-Chen Wen, and Chi-Wei Lin. 2020. A qualitative study of clinical narrative competence of medical personnel. *BMC Medical Education* 20(1):415. 10.1186/s12909-020-02336-6 33167943 10.1186/s12909-020-02336-6 PMC7653871

[CR22] Clark, Samantha. 2020. *The Clearing: A Memoir of Art, Family and Mental Health*. London: Little, Brown.

[CR23] Cooper, Steven H. 2024. Being careful in only a perverse way’: The use of aesthetic experience in psychoanalytic work. *Psychoanalytic Inquiry* 44(4):346–357. 10.1080/07351690.2024.2345569

[CR24] Dewey, John. 2005. *Art as Experience*. London: Penguin.

[CR25] Gadamer, Hans-Georg. 1986. *The Relevance of the Beautiful and Other Essays*. Cambridge: Cambridge University Press.

[CR26] Gilman, Charlotte Perkins. 2004. *Charlotte Perkins gilman’s the yellow Wall-Paper: A sourcebook and critical edition*, ed. J. Catherine, and Golden. New York: Routledge.

[CR27] Hagman, George. 2005. *Aesthetic experience: Beauty, creativity, and the search for the ideal*. Amsterdam: Brill.

[CR28] Hohendahl, Peter Uwe. 1995. *Prismatic Thought: Theodor W. Adorno*. University of Nebraska Press.

[CR29] Hohendahl, Peter Uwe. 2010. *The fleeting promise of art: Adorno’s aesthetic theory revisited*. Ithaca: Cornell University Press.

[CR30] Inhorn, Marcia C., and Lucia Volk, eds. 2021. *Un-settling middle Eastern refugees: Regimes of exclusion and inclusion in the middle East, Europe, and North America (Volume 40)*. New York: Berghahn Books.

[CR31] Jay, Martin. 2005. *Songs of experience: Modern American and European variations on a universal theme*. Berkeley: University of California Press.

[CR32] Kant, Immanuel. 1998. *Critique of Pure Reason*. Cambridge: Cambridge University Press.

[CR33] Kant, Immanuel. 2000. *Critique of the power of judgment*. Cambridge: Cambridge University Press.

[CR34] Kelly, Martina. 2024. Unalterable testimony: Aesthetic experience in poetry and medical practice. *Journal of Applied Hermeneutics*. 10.55016/ojs/jah.v2023i2023.78510

[CR35] Kontson, Kimberly L., Murad Megjhani, Justin A. Brantley, G. Jesus, Sho Cruz-Garza, Dario Nakagome, Michelle Robleto, Eugene White, Civillico, and Jose L. Contreras-Vidal. 2015. Your brain on art: Emergent cortical dynamics during aesthetic experiences. *Frontiers in Human Neuroscience*. 10.3389/fnhum.2015.00626. 10.3389/fnhum.2015.00626 PMC4649259 26635579

[CR36] Liatsos, Yianna. 2019. Rereading literary affordances in narrative medicine pedagogy. *Storyworlds* 11(2):1–25. 10.1353/stw.2019.0009

[CR37] Liljefors, Max. 2020. Knowledge Worlds Apart: Aesthetic Experience as an Epistemological Boundary Object. In *Movement of Knowledge: Medical Humanities Perspectives on Medicine, Science, and Experience – Medical Humanities Perspectives on Medicine, Science, and Experience*, ed. Kristofer Hansson and Rachel Irwin. 10.21525/kriterium.24.i.

[CR38] Macnaughton, Jane. 2011. Medical humanities’ challenge to medicine. *Journal of Evaluation in Clinical Practice* 17 (5): 927–32. 10.1111/j.1365-2753.2011.01728.x. 21851510 10.1111/j.1365-2753.2011.01728.x PMC4439737

[CR39] Mäkelä, Maria, Hanna Meretoja. 2022. Critical approaches to the storytelling boom. *Poetics Today* 43(2):191–218. 10.1215/03335372-9642567

[CR40] Malchiodi, Cathy A.. 2012. *Art Therapy and Health Care*. New York: Guilford Publications.

[CR41] Marković, Slobodan. 2012. Components of aesthetic experience: Aesthetic fascination, aesthetic appraisal, and aesthetic emotion. *I-Perception* 3 (1): 1–17. 10.1068/i0450aap. 23145263 10.1068/i0450aap PMC3485814

[CR42] Marx, Karl. 1977. *Early Writings*. London: Penguin.

[CR43] Mastandrea, Stefano, Sabrina Fagioli, and Valeria Biasi. 2019. Art and psychological well-being: Linking the brain to the aesthetic emotion. *Frontiers in Psychology*. 10.3389/fpsyg.2019.00739. 10.3389/fpsyg.2019.00739 PMC6458291 31019480

[CR44] Mohiuddin, Abdul Kader. 2023. Global conflict escalation during the Pandemic, Climate, and economic dilemmas: Healthcare sustainability challenges in conflict zones and elsewhere. *European Journal of Sustainable Development Research* 7(2):em0217. 10.29333/ejosdr/12936

[CR45] Ovaska, Anna. 2024. Toward engaged narratology: Critical and embodied close reading and social justice in a narrative medicine classroom. *Narrative Inquiry* 34(2):262–280. 10.1075/ni.24029.ova

[CR46] Peacocke, Antonia. 2023. Aesthetic Experience. *The Stanford Encyclopedia of Philosophy* (Fall 2024 Edition), ed. Edward N. Zalta, and Uri Nodelman. https://plato.stanford.edu/archives/fall2024/entries/aesthetic-experience/. Accessed 20 September 2025.

[CR47] Pearce, Marcus T., W. Dahlia, Oshin Zaidel, Martin Vartanian, Helmut Skov, Anjan Leder, Chatterjee, and Marcos Nadal. 2016. Neuroaesthetics: The cognitive neuroscience of aesthetic experience. *Perspectives on Psychological Science* 11(2):265–279. 10.1177/1745691615621274 26993278 10.1177/1745691615621274

[CR48] Richter, Gerhard. 2019. *Thinking with Adorno: The Uncoercive Gaze*. New York: Fordham University Press.

[CR49] Rieff, David. 2008. *Swimming In A Sea Of Death: A Son’s Memoir*. Melbourne: Melbourne University Publishing.

[CR50] Ross, Nathan. 2017. *The Philosophy and Politics of Aesthetic Experience: German Romanticism and Critical Theory*. Cham: Palgrave Macmillan. 10.1007/978-3-319-52304-0.

[CR51] Sajnani, Nisha, and Nils Fietje. 2023. The Jameel Arts & Health Lab in Collaboration with the WHO–Lancet Global Series on the Health Benefits of art. *Lancet* 402 (10414): 1732–34. 10.1016/S0140-6736(23)01959-1. 37738998 10.1016/S0140-6736(23)01959-1

[CR52] Shusterman, Richard. 2008. *Body consciousness: A philosophy of mindfulness and somaesthetics*. Cambridge: Cambridge University Press.

[CR53] Silberbusch, Oshrat C.. 2018. *Adorno’s Philosophy of the Nonidentical: Thinking as Resistance*. Cham: Palgrave Macmillan.

[CR54] Spiegel, Maura, and Danielle Spencer. 2016. This Is What We Do, and These Things Happen: Literature, Experience, Emotion, and Relationality in the Classroom. In *The Principles and Practice of Narrative Medicine*, ed. Rita Charon, Sayantani DasGupta, Nellie Herman, Craig Irvine, Eric R. Marcus, Edgar Rivera Colsn, Danielle Spencer, and Maura Spiegel, 37–59. Oxford: Oxford University Press.

[CR55] Stephens, John Paul. 2021. How the show goes on: Using the aesthetic experience of collective performance to adapt while coordinating. *Administrative Science Quarterly* 66(1):1–41. 10.1177/0001839220911056

[CR56] Tarnas, Maia C., Bachir Mohamed Hamze, Richard Tajaldin, Daniel M. Sullivan, and Parker. 2024. Exploring relationships between conflict intensity, forced displacement, and healthcare attacks: A retrospective analysis from Syria, 2016–2022. *Conflict and Health*. 10.1186/s13031-024-00630-4. 10.1186/s13031-024-00630-4 PMC11580498 39574192

[CR57] Thompson, James. 2023. *Care Aesthetics: For Artful Care and Careful Art*. Abingdon: Routledge.

[CR58] Tomlin, Adele. 2007. Introduction: Contemplating the Undefinable. In *Aesthetic Experience*, ed. Richard Shusterman and Adele Tomlin, 1–14. New York: Routledge.

[CR59] Valtonen, Jussi, and Elina Renko. 2025. Three doors to the house of perspective-taking and self-reflection: Experiences of guided narrator exploration for healthcare education. *Advances in Health Sciences Education: Theory and Practice (online ahead of print)*. 10.1007/s10459-025-10450-7. 10.1007/s10459-025-10450-7 40569494

[CR60] Vichnar, David. 2017. and Louis Armand. Aisthēsis. In *Oxford Research Encyclopedia of Literature*. Oxford University Press. 10.1093/acrefore/9780190201098.013.104. Accessed 30 April 2025.

[CR61] Viney, William, Felicity Callard, and Angela Woods. 2015. Critical medical humanities: Embracing entanglement, taking risks. *Medical Humanities* 41 (1): 2–7. 10.1136/medhum-2015-010692. 26052111 10.1136/medhum-2015-010692 PMC4484495

[CR62] Weigand, Rosalie, and Annika Moosmayer. 2021. Does self-reported chronic pain influence savoring of aesthetic experiences? *PLoS One* 16 (11): e0259198. 10.1371/journal.pone.0259198. 34767583 10.1371/journal.pone.0259198 PMC8589147

[CR63] Whiley, Lilith Arevshatian, and Gina Grandy. 2022. The ethics of service work in a neoliberal healthcare context: Doing embodied and ‘Dirty’ emotional labor. *Qualitative Research in Organizations and Management* 17(1):136–157. 10.1108/QROM-08-2020-2005

[CR64] Whitehead, Anne, and Angela Woods. 2016. *Edinburgh Companion to the Critical Medical Humanities*. Edinburgh: Edinburgh University Press. 27536757

[CR65] World Health Organization. 2022. Health and Care Workforce in Europe: Time to Act. Copenhagen: WHO Regional Office for Europe. https://www.who.int/europe/publications/i/item/9789289058339. Accessed 30 April 2025.

[CR66] Zuidervaart, Lambert. 1991. *Adorno’s Aesthetic Theory: The Redemption of Illusion*. Cambridge, MA: MIT Press.

